# AMP-EBiLSTM: employing novel deep learning strategies for the accurate prediction of antimicrobial peptides

**DOI:** 10.3389/fgene.2023.1232117

**Published:** 2023-07-24

**Authors:** Yuanda Wang, Liyang Wang, Chengquan Li, Yilin Pei, Xiaoxiao Liu, Yu Tian

**Affiliations:** ^1^ School of Modern Post (School of Automation), Beijing University of Posts and Telecommunications, Beijing, China; ^2^ School of Clinical Medicine, Tsinghua University, Beijing, China; ^3^ Laboratory Medicine, Guangdong Provincial People’s Hospital (Guangdong Academy of Medical Sciences), Southern Medical University, Guangzhou, China; ^4^ Vascular Surgery Department, Shanxi Bethune Hospital, Shanxi Academy of Medical Sciences, Tongji Shanxi Hospital, Third Hospital of Shanxi Medical University, Taiyuan, China

**Keywords:** antimicrobial peptides, diabetic foot, deep learning, ensemble learning, accurate screening

## Abstract

Antimicrobial peptides are present ubiquitously in intra- and extra-biological environments and display considerable antibacterial and antifungal activities. Clinically, it has shown good antibacterial effect in the treatment of diabetic foot and its complications. However, the discovery and screening of antimicrobial peptides primarily rely on wet lab experiments, which are inefficient. This study endeavors to create a precise and efficient method of predicting antimicrobial peptides by incorporating novel machine learning technologies. We proposed a deep learning strategy named AMP-EBiLSTM to accurately predict them, and compared its performance with ensemble learning and baseline models. We utilized Binary Profile Feature (BPF) and Pseudo Amino Acid Composition (PSEAAC) for effective local sequence capture and amino acid information extraction, respectively, in deep learning and ensemble learning. Each model was cross-validated and externally tested independently. The results demonstrate that the Enhanced Bi-directional Long Short-Term Memory (EBiLSTM) deep learning model outperformed others with an accuracy of 92.39% and AUC value of 0.9771 on the test set. On the other hand, the ensemble learning models demonstrated cost-effectiveness in terms of training time on a T4 server equipped with 16 GB of GPU memory and 8 vCPUs, with training durations varying from 0 to 30 s. Therefore, the strategy we propose is expected to predict antimicrobial peptides more accurately in the future.

## 1 Introduction

Antimicrobial peptides are a class of small peptide molecules widely present both inside and outside of organisms, possessing strong antibacterial and antifungal properties (Zasloff, 2002). Their mechanism of action primarily involves disrupting microbial cell membranes, leading to cell death (Brogden, 2005). The biological structure of antimicrobial peptides usually encompasses various amino acids, offering a broader antimicrobial spectrum and lower resistance than traditional antibiotics (Hancock and Sahl, 2006; Mahlapuu et al., 2016). This makes them promising candidates for applications in biomedical, food preservation, cosmetic antimicrobial, and environmental protection (Axel et al., 2016; Kumar et al., 2018; Yoon et al., 2018) fields. For instance, in the medical domain, antimicrobial peptides serve as topical anti-infective drugs, treating skin and soft tissue infections and preventing and treating hospital-acquired infections (Omardien et al., 2016; Nakatsuji et al., 2017; Lázár et al., 2018). They are also extensively employed in medical device applications, such as coatings on pacemakers, artificial joints, and dental implants, to prevent the formation of bacterial biofilms and reduce device-related infection risks (Melo et al., 2009). In clinical therapy, antimicrobial peptides have gradually attracted attention as potential alternative antibiotic treatments, demonstrating promising potential in wound healing, infectious disease treatment, and antitumor therapy (Costa et al., 2011; Hilchie et al., 2013; Mansour et al., 2014). Additionally, antimicrobial peptides have gradually attracted people’s attention as a potential alternative to antibiotic therapy and to promote the formation of new blood vessels. For example, in the clinical practice of vascular surgery, some antimicrobial peptides have been successfully used in the treatment of diabetic feet, such as LL -37 and hBDs, both of which exhibit good anti-bacterial and wound healing effects (Lázár et al., 2018; Da et al., 2021). At the same time, some studies have found that antimicrobial peptides can regulate the function of endothelial cells, pro-mote the formation of new blood vessels, and improve blood flow in the feet, thereby positively affecting the vascular lesions of diabetic feet (Xing et al., 2023).

Traditional screening methods for antimicrobial peptides include biochemical methods and molecular dynamics simulation techniques. Biochemical methods typically involve extracting peptide segments from biological samples and screening them through antimicrobial activity tests, such as the agar diffusion test and minimum inhibitory concentration determination (Hancock and Diamond, 2000; Wimley, 2010). Molecular dynamics simulations, as a bioinformatics approach, offer a new perspective for antimicrobial peptide screening. By simulating the interactions between antimicrobial peptides and bacterial target molecules, researchers can gain deeper insights into the mechanism of action of antimicrobial peptides, thereby optimizing their design and screening. Molecular dynamics simulation technology can assist researchers in screening peptide segments with higher antimicrobial activity, thereby enhancing the efficiency of antimicrobial peptide re-search and applications (Haney et al., 2017; Ulmschneider and Ulmschneider, 2018).

However, the development and screening of antimicrobial peptides currently face a series of challenges. Firstly, traditional biochemical methods are costly and have lengthy development cycles. These methods require laboratory screening of numerous peptide segments, which can consume substantial time and resources. Furthermore, due to experimental condition constraints, false-positive or false-negative results may be generated, thereby reducing the accuracy of the screening. Although molecular dynamics simulations, as a bioinformatics approach, have somewhat improved screening efficiency, they still present shortcomings. The simulation process might be con-strained by computational resources, resulting in less accurate results. Moreover, the variety of antimicrobial peptides screened might be limited, and their stability may not be sufficient to meet practical application requirements. Consequently, the development of an efficient, precise, and convenient screening strategy is crucial. Such a strategy should overcome the limitations of current screening methods, enhance screening efficiency and accuracy, and reduce research and development costs.

With the rapid advancement of AI technology and computational power, an in-creasing number of researchers have begun to focus on the identification of small functional peptides. These small peptides have shorter amino acid sequences, typically containing between 5 and 50 amino acid residues (Al-Khdhairawi et al., 2023). These short peptides play various crucial functions in biological systems, including antimicrobial, antiviral, immunoregulatory, and cellular signal transduction roles (Hancock et al., 2016). Optimized machine learning algorithms can enhance the accuracy and efficiency of identifying and predicting functional peptides, deepening our understanding of their roles in biological systems and providing robust support for related field research. Over the past few years, significant progress has been made in peptide recognition work. Meher et al. improved the accuracy of antimicrobial peptide prediction by integrating compositional, physicochemical, and structural features into the Pseudo Amino Acid Composition (PSEAAC) (Chou, 2001; Meher et al., 2017). Veltri et al. improved antimicrobial peptide identification in their research using deep learning methods (Veltri et al., 2018). Manavalan et al. enhanced prediction accuracy by using ma-chine learning and ensemble learning methods to predict cell-penetrating peptides and their engulfment efficiency (Manavalan et al., 2018). Hasan et al. proposed an improved and robust method for predicting hemolytic peptides and their activity—HLPpred-Fuse. They enhanced prediction performance by fusing various feature representations, such as amino acid composition, dihedral angles, amino acid sequence, and PSEAAC, and used a random forest (RF) for model training (Hasan et al., 2020). Although existing research has made some break-throughs in identifying antimicrobial peptides, the precision of prediction and the efficiency of screening still need improvement. These methods might encounter low computational efficiency and high time costs when handling large-scale datasets. While existing methods have contributed significantly to the identification of these peptides, there’s a need for more versatile approaches that can rapidly adapt to diverse identification requirements. Furthermore, some models’ generalization capability on new datasets needs to be strengthened. Hence, our work presents a new approach that addresses this gap, by developing a prediction model that offers flexibility and efficiency in identifying antimicrobial peptides under diverse conditions.

**FIGURE 1:**
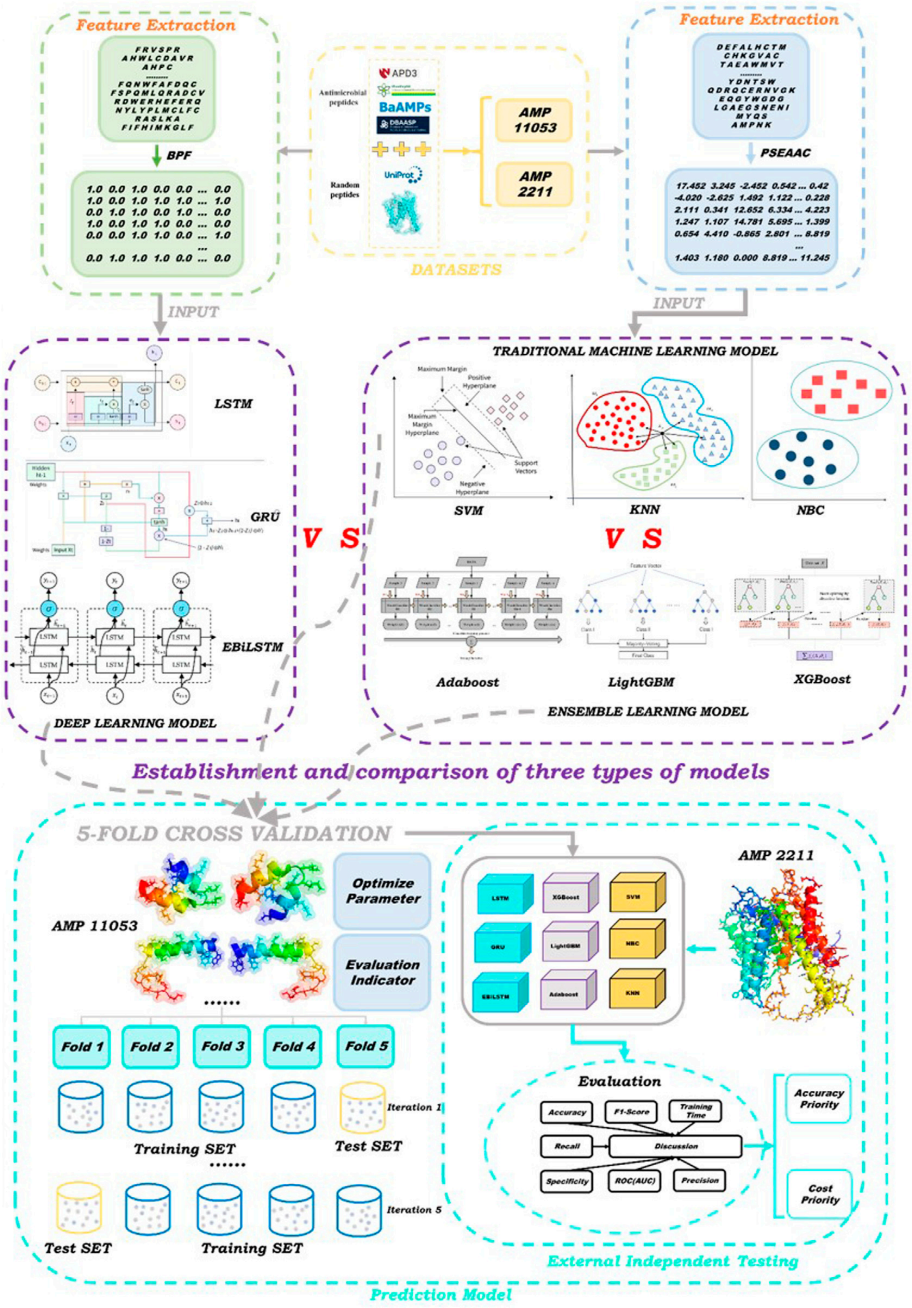
Schematic showing experimental workflow.

The aim of this study is to develop an accurate and efficient antimicrobial peptide screening strategy using novel deep learning models. We constructed two datasets: the first for training and five-fold cross-validation, and the second for external independent testing. We proposed the Enhanced Bi-directional Long Short-Term Memory (EBiLSTM) deep learning model and compared it with mainstream ensemble learning and baseline models. In particular, our model incorporates feature fusion strategies to combine different feature types and extract comprehensive characteristics from the peptide sequences. Additionally, a multi-scale convolutional layer is used to capture peptide sequence features at various scales. These modifications aim to improve the model’s ability to recognize various features within peptide sequences, thereby enhancing its predictive performance for identifying antimicrobial peptides. For ensemble learning, we utilized Adaptive Boosting (AdaBoost), Light Gradient Boosting Machine (LightGBM), and Extreme Gradient Boosting (XGBoost). In terms of deep learning, in addition to EBiLSTM, two classic deep learning models were also selected to participate in the work. Additionally, we employed three baseline machine learning models for comparison with the aforementioned six types. These models were tested on an external dataset to evaluate their performance. The specific workflow of this study is illustrated in Figure 1.

In summary, the main contributions of this study are as follows.

- This study is the first to propose EBiLSTM models for antimicrobial peptides prediction. In detail, we made suitable modifications based on the BiLSTM network structure to enhance prediction performance.
- Regarding the dataset, we independently constructed two antimicrobial peptide datasets: one for cross-validation and another for independent verification. This provides a reliable foundation for evaluating and comparing the performance of different models.
- Considering the characteristics of different models, we separately adopted two feature extraction methods: PSEAAC and Binary Profile Feature of k-spaced Amino Acid Pairs (BPF) (Chen et al., 2016). These methods are designed to maximize the potential of each model in antimicrobial peptide prediction tasks.

## 2 Materials and methods

### 2.1 Data collection

The antimicrobial peptide data used in this study are all sourced from multiple public databases, including: APD3 (https://aps.unmc.edu/about), PlantPepDB (http://14.139.61.8/PlantPepDB/index.php), BaAMPs (https://www.baamps.it/), Bio-PepDB (https://bis.zju.edu.cn/biopepdbr/), CAMP (https://webs.iiitd.edu.in/raghava/satpdb/catalogs/camp/), DBAASP (https://dbaasp.org/home), DRAMP (https://dramp.cpu-bioinfor.org/), LAMP (https://ngdc.cncb.ac.cn/databasecommons/database/id/4562). After screening, we obtained 5605 and 1119 antimicrobial peptide samples from these databases, respectively. Simultaneously, to construct a comparable proportion of negative samples to the antimicrobial peptide samples, we referred to previous studies (Tyagi et al., 2013; Kumar et al., 2015) and randomly selected the corresponding number of peptide sequences from the UniProt database. These negative samples primarily included peptides that are non-antimicrobial. We aimed to ensure a balance with our positive samples, and therefore, additional criteria were considered in their selection. We ensured that these samples were similar to our positive samples in terms of length, to prevent the length from becoming a distinguishing feature. We also took into account the amino acid composition, ensuring that the negative samples did not exhibit any uncommon composition that could introduce bias. Peptide sequences were added to two datasets, which we named AMP-11053 and AMP-2211. The AMP-11053 dataset was used for model training and internal validation (i.e., five-fold cross-validation), while the AMP-2211 dataset was used for external independent testing to evaluate the model’s generalization performance. After the construction of the datasets, we ensured that there were no duplicate peptide sequences within or between the two datasets through careful verification. This procedure helps to ensure the reliability of model training and evaluation.

### 2.2 Peptide sequence feature representation

To fully tap into the potential of different models for antimicrobial peptide identification tasks, we adopted a variety of model types in this study. Considering the characteristics of each type of model, we chose different feature extraction methods to match their respective applicability. Specifically, for ensemble learning and traditional machine learning models, we utilized the PSEAAC feature extraction method, which has demonstrated commendable performance in many bioinformatics problems. For deep learning models, we selected the BPF feature extraction method. This method effectively captures the local features of sequences, thereby enhancing the performance of the models.

#### 2.2.1 Binary profile feature of k-spaced amino acid pairs

BPF is a feature extraction method used to characterize protein sequences. It con-siders the binary representation of amino acid pairs with k intervals in the amino acid sequence, thereby capturing the relationship between locally adjacent amino acids. After determining the value of k, the BPF algorithm constructs a binary matrix with 20 × 20 rows and columns equivalent to the sequence length minus k. The matrix is populated based on the occurrence of amino acid pairs in the sequence. If a specific pair appears in the sequence, the corresponding position in the matrix is filled with 1; otherwise, it is filled with 0. The binary matrix is then flattened into a feature vector for subsequent analysis.

To determine the appropriate value of k, we extracted 15% of the data from the AMP-11053 dataset as a pre-experimental dataset and conducted pre-experiments with k set to 0, 1, 2, 3, 4, and 5, respectively. The average AUC value was calculated through five-fold cross-validation, and the AUC curve was plotted. The results showed that the AUC value was highest when k = 3, so we selected k = 3 as the parameter for the BPF method in this study. Subsequently, the AMP-11053 and AMP-2211 datasets processed using the BPF method were used as inputs for the deep learning models.

#### 2.2.2 Pseudo amino acid composition

PSEAAC is a feature extraction method widely applied in the field of bioinformatics, primarily used to represent protein sequences. This method integrates both local and global features of amino acid sequences to generate a feature vector of fixed length. Any peptide sequence can be represented as shown in Equation 1, with the specific calculation formula $x_{\mu }$ for different subscripts as given in Equation 2. Here, the integer *λ* represents the highest order of sequence correlation, and ω is a weight coefficient between 0 and 1. $f_{i}\left(i=1,2,...20\right)$ represents the frequency of occurrence of the 20 natural amino acids in the peptide, and $\theta_{j}\left(j=1,2,...,\lambda \right)$ denotes the correlation factor of order j, which is defined as shown in Equation 3. The correlation function is calculated according to Equation 4, where $X_{1}\left(R_{i}\right)$ , $X_{2}\left(R_{i}\right)$ , … $X_{n}\left(R_{i}\right)$ represent the physicochemical properties of $R_{i}$ (Ge et al., 2020).

$$P=\left[x_{1},x_{2},...x_{20},x_{20+1},...x_{20+\lambda }\right]$$ (1)

$$x_{\mu }=\left\{\begin{array}{c} \frac{f_{\mu }}{\sum_{i=1}^{20}f_{i}+\omega \sum_{j=1}^{\lambda }\theta_{j}}\left(1\leq \mu \leq 20\right)\\ \frac{\omega \theta_{\mu -20}}{\sum_{i=1}^{20}f_{i}+\omega \sum_{j=1}^{\lambda }\theta_{j}}\left(20+1\leq \mu \leq 20+\lambda \right) \end{array}\right.$$ (2)

$$\theta_{j}=\frac{1}{L-j}\sum_{i=1}^{L-j}\Theta \left(R_{i},R_{i+j}\right)\left(1\leq j\leq \lambda \right)$$ (3)

$$\Theta \left(R_{i},R_{j}\right)=\frac{1}{n}\left\{{\left[X_{1}\left(R_{i}\right)-X_{1}\left(R_{j}\right)\right]}^{2}+{\left[X_{2}\left(R_{i}\right)-X_{2}\left(R_{j}\right)\right]}^{2}+...+{\left[X_{n}\left(R_{i}\right)-X_{n}\left(R_{j}\right)\right]}^{2}\right\}$$ (4)

PSEAAC has two types: Type 1 and Type 2. In this study, we employed the Type 2 PSEAAC approach and selected six physicochemical properties, namely, ‘Hydrophobicity,’ ‘Hydrophilicity,’ ‘Mass,’ ‘pK1’ (acid dissociation constant), ‘pK2’ (base dissociation constant), and ‘pI’ (isoelectric point). In the experiment, the weight was set to 0.05, and the sequence interval (lambda) was set to 2. The processed features were then in-putted into ensemble learning models and baseline machine learning models for further analysis.

### 2.3 Deep learning model construction

#### 2.3.1 Enhancing bidirectional long short-term memory

BiLSTM is a unique variant of LSTM networks designed to consider both forward and backward information in an input sequence (Schuster and Paliwal, 1997). Traditional LSTM networks process sequence data in a forward manner, unable to capture information from future elements. However, BiLSTM enhances this by adding a parallel LSTM layer to the original, which processes the input sequence in reverse order. This bidirectional characteristic empowers the model to grasp the context of both preceding and subsequent sequences at any given point, furnishing a more comprehensive apprehension of the sequence context. Such a feature makes BiLSTM superior to traditional LSTM in tasks with bidirectional dependencies, such as part-of-speech tagging, named entity recognition, semantic role labeling, and more, offering significant advantages in the identification of antimicrobial peptides.

**FIGURE 2:**
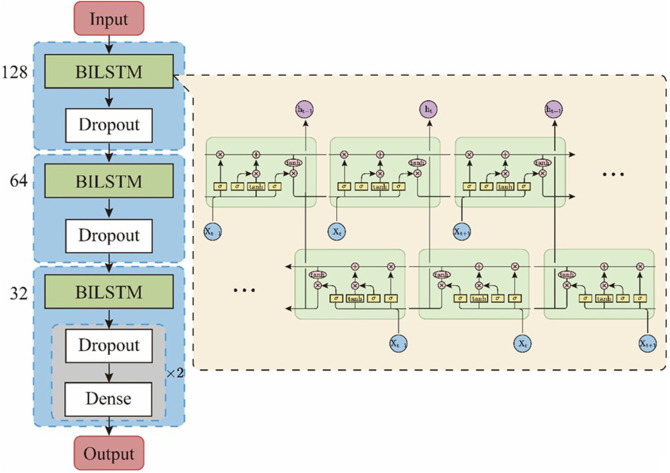
Structure of the EBiLSTM model.

In this study, we designed an EBiLSTM model composed of three BiLSTM net-works, as illustrated in Figure 2. Our model accepts input of size (100, 20), corresponding to sequence data with a length of 100 and feature dimension of 20. The model begins with a bidirectional LSTM layer containing 128 units and a dropout ratio of 0.5, followed by a dropout layer of 0.3. Subsequent bidirectional LSTM layers with 64 and 32 units, each followed by dropout layers, form a structure that reduces lay-er-by-layer and helps prevent overfitting. Finally, the model ends with two fully connected layers. The first layer contains 32 nodes and uses the ‘relu’ activation function, while the latter has 2 nodes and uses the ‘sigmoid’ activation function to predict the probability for each category. A dropout layer is also placed between these two layers. The model has proven to perform exceptionally, boasting high classification accuracy and robust performance.

#### 2.3.2 Long short term memory

Long Short-Term Memory (LSTM) is a unique form of recurrent neural network proposed by Hochreiter and Schmidhuber. It exhibits a remarkable memory capacity and is particularly adept at handling long sequence data, effectively sidestepping is-sues of “gradient vanishing” or “gradient explosion” (Hochreiter and Schmidhuber, 1997). In antimicrobial peptide recognition, LSTM has been proven to be a potent tool. For instance, Wang et al. utilized a parallel combination of Convolutional Neural Networks (CNN) and LSTM to identify anticancer peptides (Wang H. et al., 2021), and Christina Wang and colleagues employed LSTM to design short novel AMP sequences with potential antimicrobial activity (Wang C. et al., 2021). In this study, our network comprises multiple LSTM layers, which transmit outputs layer by layer to take full advantage of the depth of the model. To prevent overfitting, we introduced a dropout layer after each LSTM layer with a dropout rate set at 0.3. The network finally employs a fully connected layer with a sigmoid activation function to output the prediction results. Adam was chosen as the optimizer, with a learning rate set at 0.01, and a fixed random seed value of 50 was used to ensure consistency.

#### 2.3.3 Gate recurrent unit

The Gated Recurrent Unit (GRU) is an improved variant of the Recurrent Neural Network (RNN) proposed by Cho et al., in 2014 (Cho et al., 2014). By introducing two novel gating mechanisms - the update and reset gates, GRU effectively retains long-term dependency information and enhances model performance. Compared to LSTM’s four types of gates, the GRU’s structure is more concise, with fewer parameters and higher computational efficiency, yet its performance in various tasks is not inferior to LSTM’s. For instance, a model developed by Choi et al., which is based on GRU, successfully predicted patient diagnoses, drug prescriptions, and future disease risks (Choi et al., 2016). In designing the GRU deep learning network for this study, we followed design principles similar to those used with LSTM, ensuring that the model maintains high computational efficiency and robust performance when handling complex tasks. In our preliminary trials, these three models, each showcasing distinct strengths in managing sequence data, emerged as the superior performers in predicting antimicrobial peptides. Consequently, we selected them for our research.

### 2.4 Ensemble learning model construction

#### 2.4.1 Adaptive boosting

AdaBoost is a powerful ensemble learning technique, central to which is the concept of integrating multiple weak classifiers to enhance model performance (Freund and Schapire, 1997). In bioinformatics, as shown in research by Haoyi Fu et al., AdaBoost has been successfully applied to identify the structure and physicochemical properties of antimicrobial pep-tides (Fu et al., 2020). We chose Adaboost for its remarkable capability to concentrate on challenging-to-classify instances by progressively emphasizing the data misclassified by the preceding classifier. In this study, we used a decision tree as the base classifier, set an iteration limit of 200 to avoid overfitting, controlled the step size of the training process with a learning rate of 0.05, and selected ‘SAMME.R’ as the algorithm scheme to achieve genuine boosting effects. To ensure the consistency of the experimental results, we set a fixed random seed value of 50. These settings allowed our AdaBoost classifier to achieve a good balance in terms of robustness and stability.

#### 2.4.2 Light gradient boosting machine

LightGBM, developed by Microsoft Research (Ke et al., 2017), is an efficient and accurate gradient boosting decision tree algorithm characterized by its rapid training speed and low memory usage. It employs a histogram-based gradient boosting technique and a leaf-wise growth strategy, effectively enhancing training speed and optimizing the handling of imbalanced data, it is also recognized for its superior accuracy, a critical attribute essential for our study. In our study, key parameters were set as follows: ‘num_leaves’ was set to 20 to control model complexity and prevent overfitting; ‘min_data_in_leaf’ was also set to 20 to further guard against overfitting; the depth of the decision tree was unrestricted; the learning rate was set at 0.3 to ensure a balance between training speed and performance; 100 trees were used for fitting; a binary loss function was selected; the traditional gradient boosting decision tree method was employed; and the random seed was set to 40 to ensure the reproducibility of the experiment.

#### 2.4.3 Extreme gradient boosting

XGBoost is an advanced algorithm centered around gradient boosting decision trees, developed by Chen et al. (Chen and Guestrin, 2016). It is highly acclaimed for its superior predictive power and efficient computational speed. By using the second-order derivative information of the objective function and a regularization term, XGBoost optimizes predictive accuracy. Furthermore, by introducing column block data storage and performing parallel and distributed optimizations, it greatly enhances computational efficiency. By utilizing a more regularized model formulation to curb overfitting, it demonstrates superior performance over other models across a range of datasets. XGBoost has also been applied in the medical field; for instance, Junjie Huang et al. utilized it in their machine learning pipeline to identify potent antimicrobial peptides across the entire peptide sequence space (Huang et al., 2023). In our study, the CART tree was chosen as the base learner, and the maximum depth of weak learners and the maximum number of trees were set to 6 and 10, respectively, to prevent overfitting. The learning rate was set to 0.1 to control the step size of iterative updates, and the subsample ratio was set to 0.2 to enhance the model’s generalization ability. Additionally, the random seed value was set to 50 to enhance model stability. These settings enabled the XGBoost model to achieve excellent results in terms of predictive performance and stability.

### 2.5 Baseline model

To comprehensively evaluate the performance of our models, we chose to com-pare them against traditional machine learning models often used in small peptide screening, such as the Support Vector Machine (SVM), Naive Bayes Classifier (NBC), and K-Nearest Neighbors (KNN) (Manavalan et al., 2017; Khabbaz et al., 2021; Wani et al., 2021; Jiang et al., 2022). For SVM, we employed the Gaussian radial basis function kernel to address non-linear classification problems, set the C parameter to 1.0 to balance misclassification penalties, enabled the probability option to output prediction probabilities, and allowed the model to optimize the gamma parameter automatically. We used Gaussian Naive Bayes as it assumes that the continuous features follow a Gaussian distribution. For KNN, we set the number of neighbors, k, to 5 to balance bias and variance and used Euclidean distance as the metric. By comparing these traditional models, we further validated the performance and robustness of our deep learning and ensemble learning models.

### 2.6 Experiment

In this study, we adopted the widely accepted method of five-fold cross-validation for model training on the AMP-11053 dataset. This approach divides the dataset into five portions, with four of them being used for training and the remaining one for validation. By alternating the training and validation sets, five rounds of training and validation were conducted, with the final model performance evaluation result being the average of the five validation results. Throughout the model training process, we performed parameter optimization on all models to achieve optimal performance. On the AMP-2211 dataset, we carried out independent testing to further validate the models’ generalization capability. The experimental environment was configured as follows: we used a T4 server with 16 GB of GPU memory and 8 vCPUs, equipped with 32 GB of RAM, running on a Linux operating system. We utilized the Python 3.8 programming language for model writing and training, relying on machine learning libraries such as Tensorflow 2.2.0 and Scikit-learn 1.2.2 for the construction of deep learning models and implementation of traditional machine learning models. This setup strikes a balance between abundant computational resources and the use of common, easily accessible hardware devices, aiming to ensure the replicability of our study’s results.

### 2.7 Model evaluation

To comprehensively assess model performance, we adopted metrics such as Ac-curacy, Recall, Specificity, Precision, F1-Score, and AUC value, and also plotted ROC curves (Bradley, 1997; Sokolova and Lapalme, 2009; Powers, 2020). TP, TN, FP, FN in the confusion matrix are the primary evaluation parameters, representing true positives, true negatives, false positives, and false negatives. Accuracy calculates the proportion of samples that the model correctly predicts, Precision measures the proportion of true positive samples in those predicted as positive, while Specificity reflects the proportion of true negative samples that were correctly predicted. The F1-score is the harmonic mean of precision and recall. Through the ROC curve, we can see the classifier’s performance under all possible classification thresholds, and the area under the curve (AUC) quantifies the overall performance of the classifier. The closer the AUC value is to 1, the better the model performance. In detail:

$$Accuracy=\frac{TP+TN}{TP+TN+FP+FN}$$ (5)

$$Precision=\frac{TP}{TP+FP}$$ (6)

$$Specificity=\frac{TN}{TN+FP}$$ (7)

$$Recall=\frac{TP}{TP+FN}$$ (8)

$$F1-score=\frac{2\times Precision\times Recall}{Precision+Recall}$$ (9)

## 3 Results

### 3.1 Statistical results of amino acids in the dataset

**FIGURE 3:**
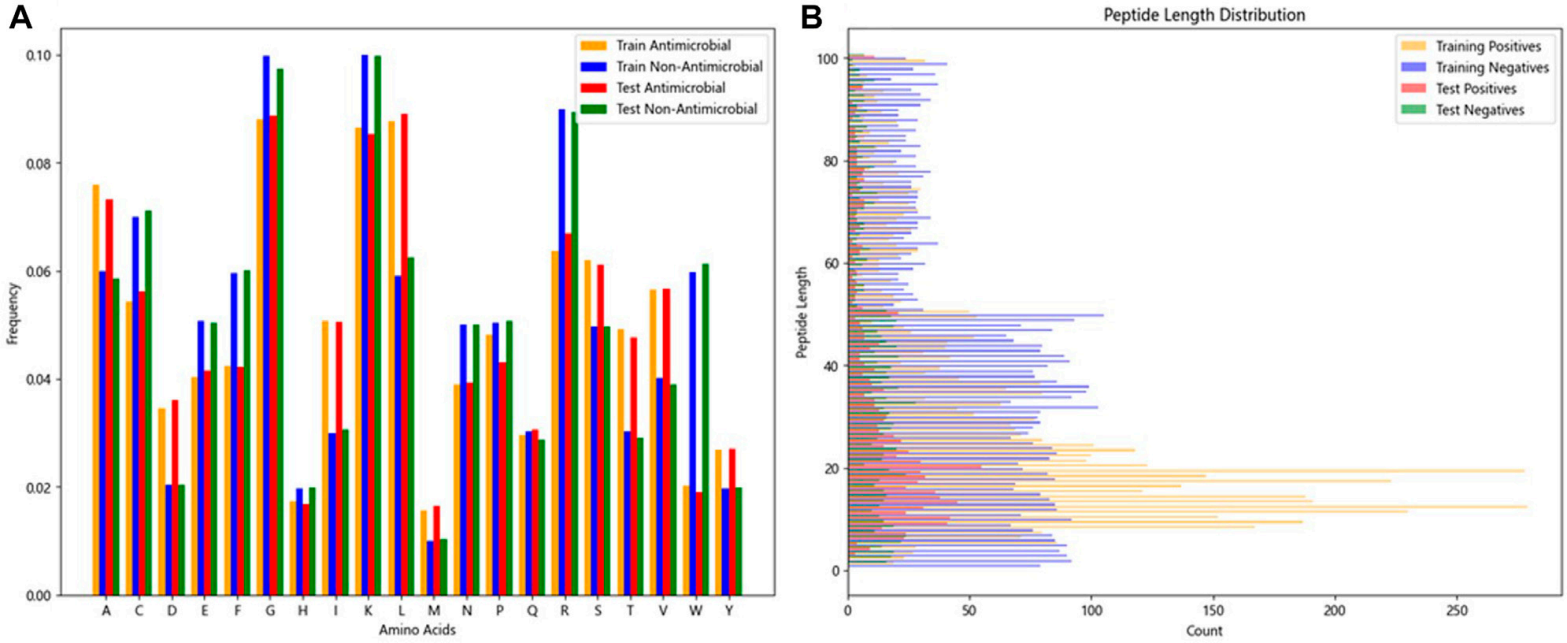
Features of peptide chains from two datasets.**(A)** Frequency distribution ratio of various amino acids in peptides.**(B)** Distribution of peptide lengths.

The two research datasets, AMP-11053 and AMP-2211, encompass amino acid sequences of AMPs and non-antimicrobial peptides, incorporating 20 common natural amino acids. Figure 3A depicts the distribution of amino acid frequencies in AMPs and non-AMPs across both datasets. Upon close inspection, we can observe a degree of similarity in the distribution of amino acid frequencies between AMPs and non-AMPs, which not only reflects the complexity of the classification task but also underscores the challenges and value of this research. Furthermore, the analysis of sequence length distribution between AMPs and non-AMPs is shown in Figure 3B, with most AMP sequence lengths falling between 5 and 50 amino acids. Similarly, non-AMP sequences also have a rich distribution within this length range.

### 3.2 Deep learning model results

**FIGURE 4:**
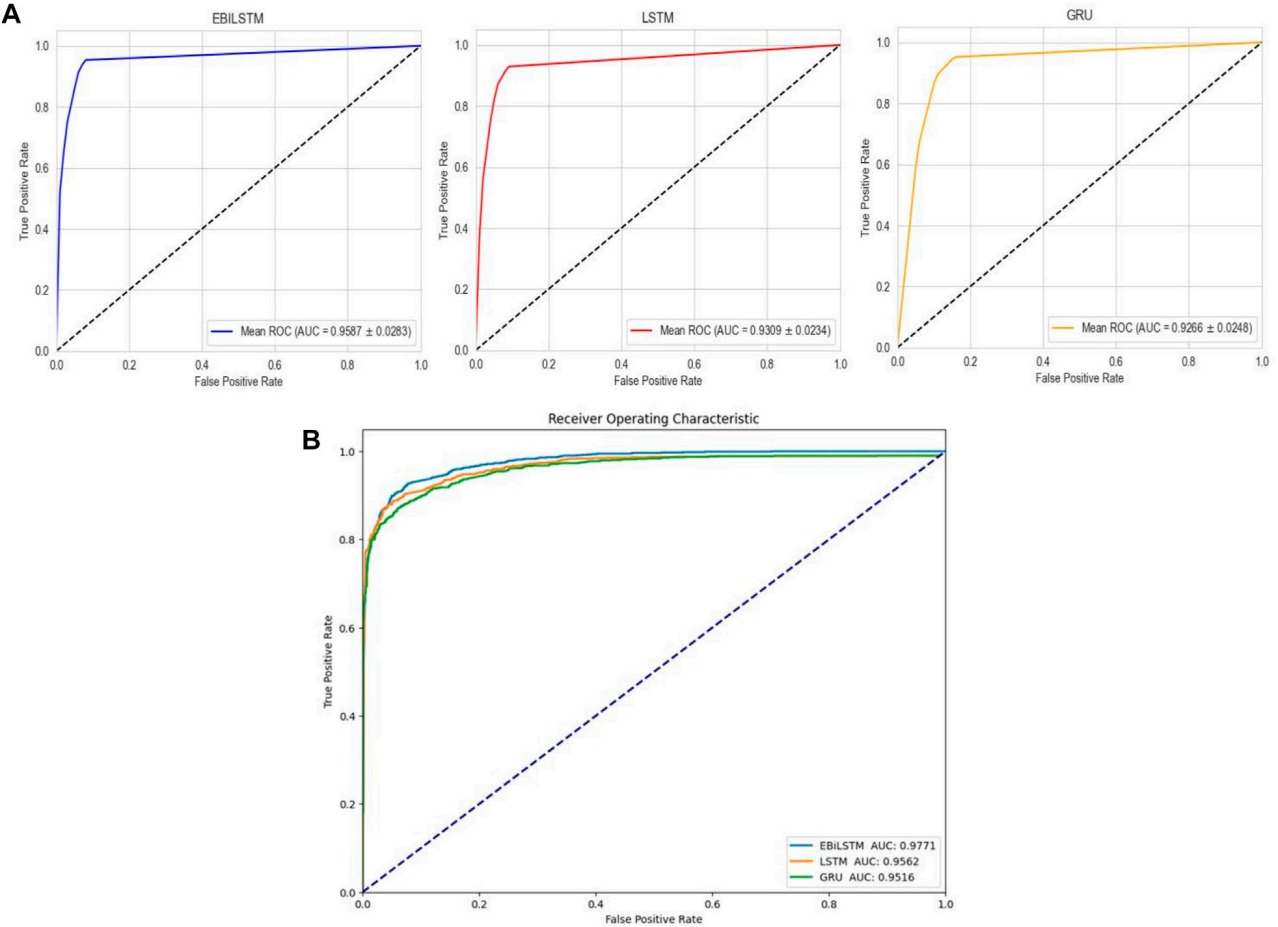
Performance of the deep learning models on datasets. **(A)** ROC curves and AUC values of EBiLSTM, LSTM, and GRU after 5-fold cross-validation on the AMP-11053 dataset. **(B)** ROC curves and AUC values of EBiLSTM, LSTM, and GRU on the validation set AMP-2211.

In the experiments conducted on the AMP-11053 dataset, we utilized LSTM, GRU, and EBiLSTM as models for training. During the training process, each model was trained 500 times. We employed multi-class logarithmic loss as the loss function, and the accuracy served as the evaluation standard. The training strategy involved five-fold cross-validation to ensure more stable and reliable model evaluations. We implemented an early termination criterion which stops the training process if there’s no improvement in the validation set performance over a defined number of epochs. This strategy not only conserves computational resources, but also aids in preventing the model from assimilating noise present in the training data. Simultaneously, we also computed the evaluation metrics mentioned in Section 3.1. The training results, which include the values of each evaluation metric, the AUC curve, and the ROC values, are shown in Table 1 and Figure 4A. Similarly, we tested the models’ generalization capabilities on an additional external dataset, AMP-2211. The testing results are presented in Table 2 and Figure 4B. It is not difficult to find that the proposed EBiLSTM has the most excellent performance both in the training set and the external test set.

**TABLE 1:** Performance of the deep learning models on AMP-11053.

AMP-11053	Accuracy	Recall	Specificity	Precision	F1-score
EBILSTM	0.9685 ± 0.0408	0.9619 ± 0.0426	0.9654 ± 0.0388	0.9663 ± 0.0376	0.9699 ± 0.0401
LSTM	0.9383 ± 0.0329	0.9139 ± 0.0472	0.9558 ± 0.0207	0.95640 ± 0.0213	0.9375 ± 0.0334
GRU	0.927 ± 0.0512	0.9489 ± 0.0097	0.9043 ± 0.1004	0.912 ± 0.0768	0.9265 ± 0.0440

**TABLE 2:** Results derived from the independent external validation set, AMP-2211.

AMP-2211	Accuracy	Recall	Specificity	Precision	F1-score
EBILSTM	0.9239	0.9186	0.9294	0.9303	0.9244
LSTM	0.9099	0.8971	0.9132	0.9217	0.9189
GRU	0.9018	0.9045	0.8846	0.8907	0.9044

While our models achieved excellent performance on the AMP-11053 dataset, as evidenced by the evaluation metrics, AUC curve, and ROC values in Table 1 and Figure 4A, the performance on the external dataset AMP-2211, depicted in Table 2 and Figure 4B, was marginally lower. It is crucial to note that this dip in performance, while important to acknowledge, is not entirely unexpected. When applying a model trained on one dataset (AMP-11053) to a different dataset (AMP-2211), it is common to see some decrease in performance. This is due to the inherent differences between the datasets, which might include variations in complexity, distribution of data, or the amount and type of noise present. Essentially, the AMP-2211 dataset presents previously unseen scenarios for the model, and it is natural that the model will not perform as effectively on this new data as on the data it was trained on. However, this difference in performance can actually be seen as a positive. If our model performed identically on both datasets, it would raise concerns about overfitting. Overfitting occurs when a model learns the training data too well, to the point where it is too specialized to the training data and performs poorly on new, unseen data. The fact that our model’s performance decreases slightly on the external AMP-2211 dataset suggests that our model is not overfitted and is capable of generalizing to new data.

### 3.3 Ensemble learning model results

**FIGURE 5:**
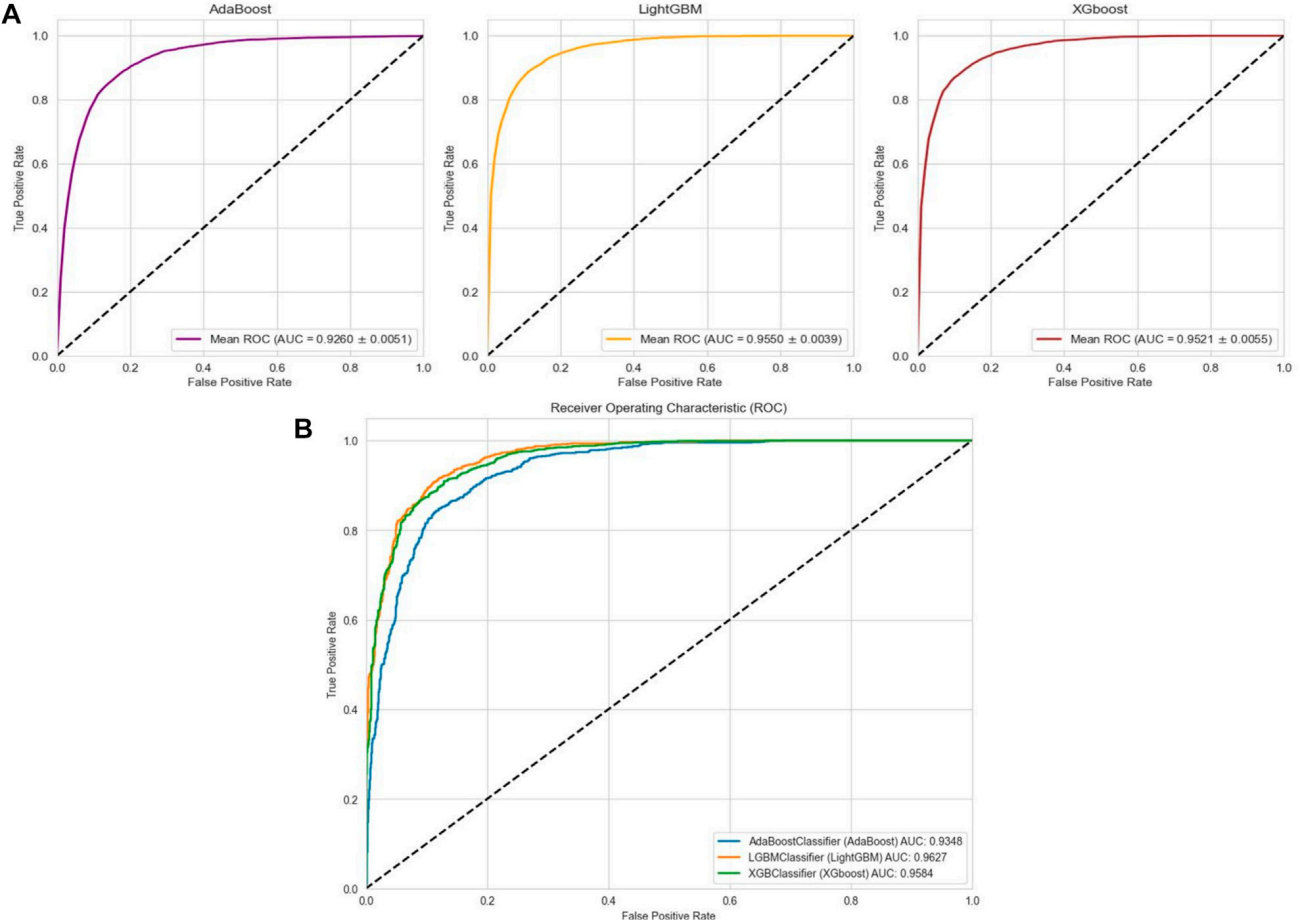
Performance of the ensemble learning models on datasets. **(A)** ROC curves and AUC values of Adaboost, LightGBM, and XGBoost after 5-fold cross-validation on the AMP-11053 dataset. **(B)** ROC curves and AUC values of Adaboost, LightGBM, and XGBoost on the validation set AMP-2211.

In the case of the AMP-11053 dataset, we trained using Adaboost, LightGBM, and XGBoost, employing a five-fold cross-validation method. We calculated five main evaluation metrics: accuracy, recall, specificity, precision, and F1-Score. During the process of evaluating model performance, to accurately assess model capabilities, we also calculated the 95% confidence interval for these metrics. Specific details are shown in Table 3, while the AUC curves derived from the three types of models are depicted in Figure 5A. To further validate the models’ generalization capabilities, we employed an additional external dataset, AMP-2211, to test the models. In the testing process, we calculated the aforementioned five evaluation metrics and drew the AUC curve. Test results are displayed in Table 4 and Figure 5B. These results provide us with a comprehensive and in-depth understanding, allowing us to assess and compare the performance of different models on multiple levels. The results above indicate that while ensemble learning demonstrates considerable accuracy in identifying antimicrobial peptides, its performance is still not on par with that of deep learning, especially EBiLSTM.

**TABLE 3:** Performance of the ensemble learning models on AMP-11053.

AMP-11053	Accuracy	Recall	Specificity	Precision	F1-score
AdaBoost	0.8432 ± 0.0095	0.8493 ± 0.0118	0.8366 ± 0.0173	0.8425 ± 0.0145	0.8459 ± 0.0108
LightGBM	0.8912 ± 0.0062	0.9058 ± 0.0089	0.8759 ± 0.0153	0.8826 ± 0.0118	0.8940 ± 0.0061
Xgboost	0.8932 ± 0.0086	0.9033 ± 0.0081	0.8824 ± 0.0123	0.8879 ± 0.0064	0.8955 ± 0.0070

Notes: The above results show the average value of each indicator and the corresponding 95% confidence interval.

**TABLE 4:** Performance of the ensemble learning models on AMP-11053.

AMP-2211	Accuracy	Recall	Specificity	Precision	F1-score
AdaBoost	0.8408	0.8365	0.8452	0.8471	0.8417
LightGBM	0.8996	0.9097	0.8892	0.8938	0.9017
Xgboost	0.9032	0.9133	0.8929	0.8973	0.9052

### 3.4 Baseline model results

**FIGURE 6:**
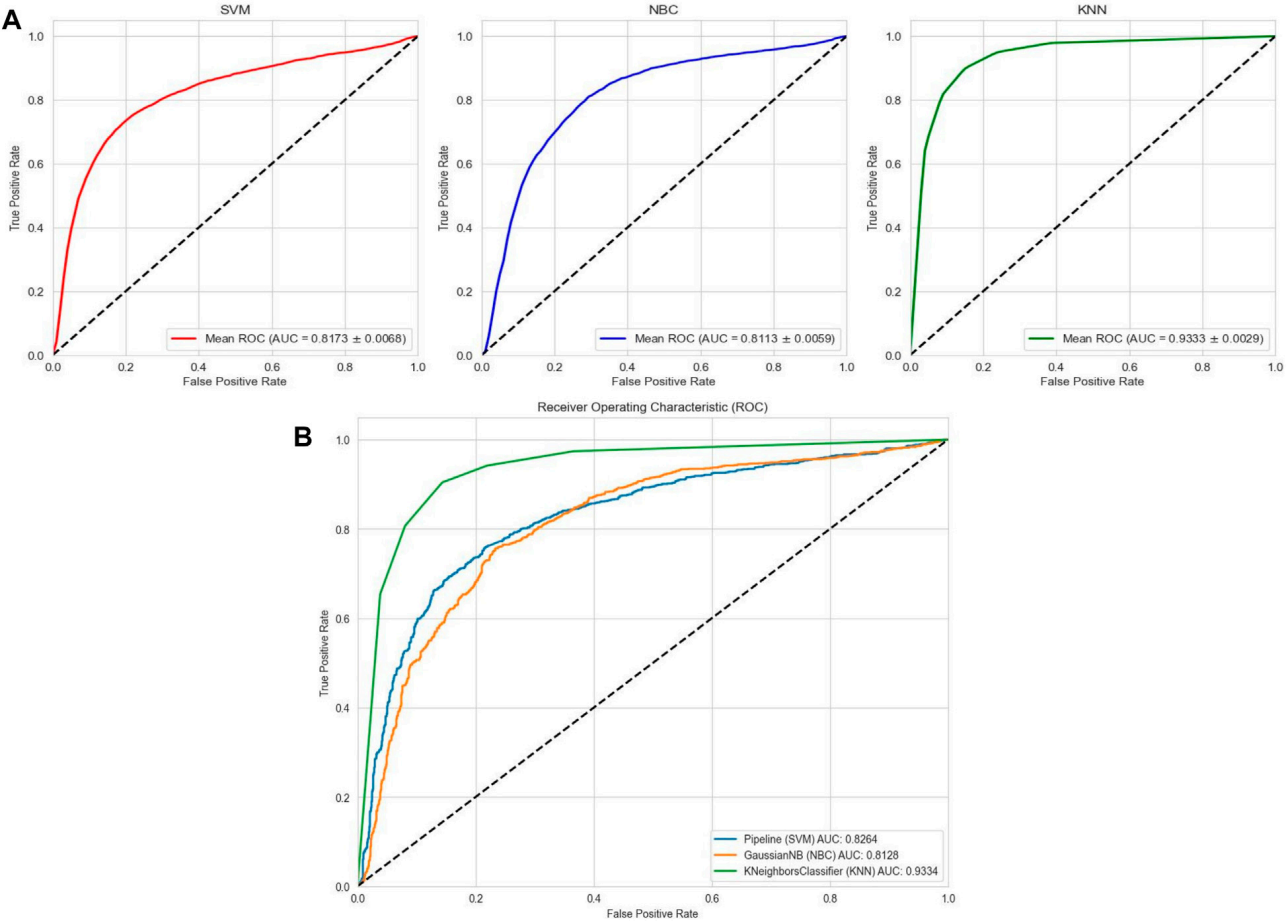
Performance of traditional machine learning models on datasets. **(A)** ROC curves and AUC values of SVM, KNN, and NBC after 5-fold cross-validation on the AMP-11053 dataset. **(B)** ROC curves and AUC values of SVM, KNN, and NBC on the validation set AMP-2211.

To comprehensively validate the performance of our models, we used traditional machine learning models, SVM, NBC, and KNN, as benchmarks for comparison with the two categories of models mentioned earlier. On the AMP-11053 dataset, the results from the five-fold cross-validation of the traditional models are shown in Table 5, with the specific AUC curves and ROC values illustrated in Figure 6A. We also evaluated the generalization capabilities of each model on an external dataset, AMP-2211. The results of these external validations are listed in Table 6 and depicted in Figure 6B. Among them, K-NN performs the best, but its performance is still not as good as the prediction strategy proposed above.

**TABLE 5:** Performance of the traditional machine learning models on AMP-11053.

AMP-11053	Accuracy	Recall	Specificity	Precision	F1-score
SVM	0.7663 ± 0.0071	0.7309 ± 0.0057	0.8030 ± 0.0139	0.7919 ± 0.0277	0.7601 ± 0.0140
NBC	0.7167 ± 0.0160	0.5617 ± 0.0173	0.8763 ± 0.0088	0.8234 ± 0.0211	0.6678 ± 0.0169
KNN	0.8749 ± 0.0066	0.8997 ± 0.0068	0.8494 ± 0.0066	0.8597 ± 0.0154	0.8792 ± 0.0097

**TABLE 6:** Evaluation outcomes from the external standalone validation dataset, AMP-2211.

AMP-2211	Accuracy	Recall	Specificity	Precision	F1-score
SVM	0.7698	0.7623	0.7775	0.7783	0.7702
NBC	0.7092	0.5416	0.881	0.8234	0.6534
KNN	0.8806	0.9044	0.8562	0.8657	0.8846

## 4 Discussion

In this study, we have focused on finding accurate strategies for antimicrobial peptide screening. With an emphasis on ensemble learning and deep learning methods, we constructed two datasets which were applied for model training, cross-validation, and independent external testing. This ensured rigorous and impartial model evaluation. By comparing various evaluation metrics, we analyzed the performance of the ensemble learning and deep learning models in prediction tasks. The results revealed that our custom-built EBiLSTM model had the highest accuracy, nearly 98% on the test set, demonstrating its significant predictive power in this prediction tasks. In further analyses, our EBiLSTM model was not only effective in peptide screening, but also efficient, significantly reducing the time and resources needed for conventional experimental methods. These results illustrate the potential utility of ensemble learning and deep learning methods in biomolecular studies. The success of the EBiLSTM model underscores the power of these algorithms in handling complex biological data and has promising implications for accelerating antimicrobial peptide discovery. Going forward, we plan to improve this model by integrating additional features and refining hyperparameters to further enhance its predictive capacity. Our ultimate aim is to contribute to effective solutions against antibiotic resistance.

Deep learning outperforms both traditional machine learning and ensemble learning models in terms of accuracy. To understand why the EBiLSTM model exhibits optimal performance, it is necessary to analyze the network architecture. Firstly, from a design perspective, the model incorporates three BiLSTM layers and four dropout layers. The multi-layer network structure equips the model with sufficient capacity to learn complex patterns in the sequence. An appropriate dropout rate (the optimal value of 0.3, chosen after numerous experiments) plays a key role in preventing over-fitting and enhancing the model’s generalization capability. In the final fully connect-ed layer, the network adopts a ReLU activation function. ReLU alleviates the vanishing gradient problem, thereby enhancing the model’s learning ability. Concurrently, the number of neurons in each BiLSTM layer is judiciously halved, maintaining sufficient model complexity while avoiding the issue of low computational efficiency. Examining model specifics, most operations in the EBiLSTM are point-wise, such as the activation functions of various gates and the update of cell states. The advantage of these point-wise operations is their high degree of parallelism, enabling the model to effectively utilize the parallel computational capabilities of modern hardware, thus achieving high efficiency in practical applications.

Ensemble learning models and traditional machine learning models have been used extensively in various applications due to their simplicity and interpretability. However, when it comes to predicting AMPs, these models have several limitations. Firstly, they typically operate on a feature-engineering basis, where appropriate features need to be manually extracted from the peptide sequences. This can often be a time-consuming process and may overlook complex patterns or dependencies in the data that could be critical to accurate prediction. Secondly, these models usually treat sequences as fixed-length inputs and lose valuable information when sequences are of variable lengths. This is a significant challenge as peptides can have different lengths, and disregarding this variation can lead to sub-optimal predictions. Finally, these models lack the capacity to automatically learn and improve from data in the same way that deep learning models can. They do not adapt their structure and parameters based on the complexity of the task at hand, which can lead to lower prediction accuracy. In contrast, deep learning models, like our proposed AMP-EBiLSTM, can automatically extract features, accommodate variable-length sequences, and improve over time by learning intricate data patterns. As such, they can often outperform ensemble and traditional machine learning models in complex predictive tasks such as AMP prediction.

Training cost is a key consideration in the application of machine learning and deep learning. Compared to deep learning models, baseline machine learning and ensemble learning models have lower training costs. Ensemble learning and baseline models exhibit low costs in terms of training time, with the training time ranging from 0 to 30 s on our equipment. From the perspective of the number of model parameters, baseline machine learning and ensemble learning models usually have significantly fewer parameters than deep learning models. Secondly, in terms of the training process, baseline machine learning models are typically more concise and efficient. Specifically, SVM is based on the solution of convex optimization problems, NBC is grounded in statistical theory of conditional probability, KNN is based on distance measurement, while Adaboost, LightGBM, and XGboost are implemented through the iterative optimization of a series of weak learners. These processes are typically more efficient than complex training procedures in deep learning, such as backpropagation and gradient descent. Accurate prediction ability and low training cost may provide strong support for the early promotion of antimicrobial peptides to clinical practice. For example, the challenges faced by the application of antimicrobial peptides in clinical diseases such as diabetic foot are high production cost, poor stability, and toxicity problems. Peptides are widely used in clinical departments such as vascular surgery to provide support.

While our study presents promising outcomes, certain limitations need to be acknowledged, and potential avenues for future research should be highlighted. Firstly, despite the comprehensive dataset employed for model training and validation in this study, future research would benefit from the expansion of these datasets. To further ascertain the robustness and generalizability of our approach, it would be beneficial to accumulate more data pertaining to antimicrobial peptides and validate our models on datasets of larger scale and diversity. Secondly, although deep learning models demonstrated superior predictive performance in our study, their substantial training costs pose a challenge. Future efforts should be concentrated on refining these models to lessen training costs whilst sustaining their high predictive accuracy. This might necessitate intensive research and exploration into model architecture, training strategies, and optimization algorithms, among other aspects. Lastly, the current study has primarily focused on the theoretical screening of antimicrobial peptides. An exciting direction for future research would involve integrating our approach with wet lab experiments to provide a more precise validation of the screening results. Such empirical validation could not only further substantiate the effectiveness of our screening strategy but also assist us in comprehending and enhancing our model’s predictive out-comes, thereby bolstering the precision and efficiency of antimicrobial peptide screening. In conclusion, these identified avenues for future research will facilitate a deeper understanding and application of machine learning and deep learning in antimicrobial peptide screening. These advancements will undoubtedly contribute to bolstering the research and development of antimicrobial peptides.

## 5 Conclusion

In this study, we explored the application of deep learning techniques in con-structing models for the identification of antimicrobial peptides, aiming to strike an effective balance between wet lab experimental methods and computational predictions. We proposed a novel deep learning model- EBiLSTM, and conducted meticulous parameter tuning and comprehensive performance evaluations. The results demonstrated that although this model bore a relatively high training cost, it achieved an ac-curacy of 92.39% on the test set, with an AUC value nearing 0.98, showcasing its superior predictive performance. Our study offers fresh perspectives and possibilities for antimicrobial peptide prediction and screening. It showcases the advantages of deep learning and ensemble learning in addressing practical needs and resource conditions with flexibility, providing new research directions and tools for future studies on antimicrobial peptides.

## Data Availability

Publicly available datasets were analyzed in this study. This data can be found here: The antimicrobial peptide data used in this study are all sourced from multiple public databases, including: APD3 (https://aps.unmc.edu/about), PlantPepDB (http://14.139.61.8/PlantPepDB/index.php), BaAMPs (https://www.baamps.it/), Bio-PepDB (https://bis.zju.edu.cn/biopepdbr/), CAMP (https://webs.iiitd.edu.in/raghava/satpdb/catalogs/camp/), DBAASP (https://dbaasp.org/home), DRAMP (https://dramp.cpu-bioinfor.org/), LAMP (https://ngdc.cncb.ac.cn/databasecommons/database/id/4562).
